# The Use of Biosensors to Explore the Potential of Probiotic Strains to Reduce the SOS Response and Mutagenesis in Bacteria

**DOI:** 10.3390/bios8010025

**Published:** 2018-03-16

**Authors:** Vladimir Anatolievich Chistyakov, Evgeniya Valer’evna Prazdnova, Maria Sergeevna Mazanko, Anzhelica Borisovna Bren

**Affiliations:** Academy of Biology and Biotechnologies, Southern Federal University, 344090 Rostov-on-Don, Russia; vladimirchi@sfedu.ru (V.A.C.); mmazanko@sfedu.ru (M.S.M.); abbren@sfedu.ru (A.B.B.)

**Keywords:** *E. coli* biosensors, probiotic, antimutagens

## Abstract

A model system based on the *Escherichia coli* MG1655 (pRecA-lux) Lux-biosensor was used to evaluate the ability of the fermentates of eight probiotic strains to reduce the SOS response stimulated by ciprofloxacin in bacteria and mutagenesis mediated by it. Preliminary attempts to estimate the chemical nature of active components of the fermentates were conducted.

## 1. Introduction

Inhibitors of SOS response are potential tools for finding solutions to the problem of antibiotic resistance, as far as such resistance is often a result of the activation of the SOS-response (SOS-mediated DNA repair) followed by mutagenesis in bacteria. The therapy using bactericidal agents often stimulates SOS response [1,2]. It is shown that SOS-deficient strains adapt more slowly to antibiotics [3]. Furthermore, as has been shown in our studies [4], activation of the SOS response by one therapeutic agent can lead to acquiring resistance to another.

It seems a promising approach then to reduce antibiotic resistance (or at least to slow down the evolution of corresponding genetic factors) by switching off the SOS response in pathogenic bacteria. That is why it is necessary to look for substances that can block the activity of the genes and factors associated with the SOS pathway.

A large-scale screening, as well as developing potential inhibitors are currently underway [2,3,5,6,7,8,9]. Several promising substances have been found, such as suramin, phthalocyanine tetrasulfonic acid, etc. However, their bioavailability and cytotoxicity in eukaryotes is still questionable.

Across a number of papers, the RecA protein is considered as a primary target for inhibition as for DNA repair and other processes [1,2,9]. Therefore, most of these inhibitors were aimed at deactivating the RecA protein.

Taking into account that bacteria in multi-species communities produce a variety of substances with bactericidal activity, there should be some protective mechanisms on both sides of the barrier of interspecific competition. Just as there are natural mechanisms of resistance to antibiotics, which appeared long before mankind began to use these substances, there must be a natural mechanism that can increase the sensitivity of pathogenic bacteria to natural antimicrobials and prevent the development of resistance to them. In other words, the idea of using SOS-inhibitors in a pharmacological ensemble with antibiotics, as proposed by Alam and others [2], likely already exists in nature.

This study’s main objective was to estimate the ability of substances produced by probiotic strains to inhibit the SOS response in bacteria.

Studies concerning the newest RecA inhibitors rarely deal with their in vivo activity or their influence on the evolution of factors of antibiotic resistance. To conduct a fast and simple in vivo test, we used both a biosensor test and a mutagenesis assay confirming the most significant effects. 

Biosensor methods have not yet been widely used in studying the properties of probiotics, but recently, this situation has changed [10]. The screening based on in vivo studies on animal objects is quite common, but to perform fast screening of potential SOS-inhibitors, much simpler and less expensive in use model systems, such as biosensors, are required. 

There is a history of attempts to apply biosensors based on eukaryotic cells in the screening of probiotics [11,12]. However bacteria are, definitely, more fast-growing and easy-to-handle objects, compared to a culture of eukaryotic cells. A luminescent signal is one of the most easily detected, and that makes luminescent biosensors a tool of choice in bacterial biosensor studies. 

We use bacterial biosensors based on *E. coli* in screening for potential inhibitors of SOS-response. The Lux-biosensor model consists of *Escherichia coli* cells harboring a hybrid plasmid with two basic elements: a regulatory region (promoter and operator) and a reporter gene(s). Reporter luxCDABE genes, isolated from the genomes of luminescent bacteria and encoding luciferase and reductase, are used. As for the regulatory elements, a promoter of the *recA* gene is used. This operon is responsible for bioluminescence and provides luciferase used in this test as a reporter [13]. Thus, the increase in luminescence, recorded by the luminometer, signals about an increase in the expression of the SOS response genes.

In this system, we can identify the inhibition of the RecA pathway at the step of transcription of *RecA* because luminescence is linked to the expression of the *recA* promoter. Furthermore, we can see inhibition at other checkpoints because in addition to the construct with Lux genes, the cells of the biosensor strain also carry a normal *recA* gene in their chromosome.

Previously, we used this method to estimate the DNA-protective and antioxidant activity of bacterial fermentates [14]. Additionally, the SOS-inhibitory activity of a novel synthetic compound, nitrobenzoxadiazole derivative, 7-(1-methyl-3-pyrrolyl-)-4,6-dinitrobenzofuroxan, was shown in one of our works [4]. 

Ciprofloxacin was used as an inducer because it is known to activate the SOS response [15]. Ciprofloxacin, as well as other antibacterial quinolones, interferes with topoisomerase II. This interference leads to double-stranded DNA breaks and stalled replication forks, both of which induce the SOS-response [3].

## 2. Materials and Methods

### 2.1. Strains

Cultures of *Bacillus amyloliquefaciens* B-1895 and *Lactobacillus acidophilus* 2647 were obtained from the collection of the laboratory of experimental mutagenesis. *L. plantarum* B3242, *L. lactis* B6453, *Lactobacillus* sp. B6367, *Lactobacillus* sp. B6379 and *L. rhamnosus* were kindly provided by V.G. Melnikov, Moscow Research Institute of Epidemiology and Microbiology. *Bacillus subtilis* KATMIRA1933 was provided by prof. M.L. Chikindas, Rutgers State University.

### 2.2. Preparation of Fermentates

Growing conditions of bacilli strains and the method of obtaining supernatants was similar to that used in our previous work [14] Lactobacilli strains were grown in a medium containing ultra-pasteurized milk, the culture of which was looped and cultured for two days at a temperature of 36 °C. Then, by adding 0.1 mM NaOH solution, the pH was adjusted to a neutral level so that acidity would not affect the functioning of biosensors. 

### 2.3. Biosensor Test

*Escherichia coli* strain MG1655 (*pRecA*-lux) (obtained from Manukhov, State Scientific Center Genetika, Moscow, Russia) was used as a Lux biosensor, identifying induction of the RecA promoter, which is involved in SOS-reparation. In addition to this construct, the plasmid also carries an ampicillin resistance gene. The biosensor strain was grown in Luria–Bertani (LB) broth (Difco, Detroit, MI, USA) at 37 °C.

SOS-inhibitive activity was evaluated by the ability of studied metabolites to reduce the SOS-response, stimulated by ciprofloxacin at concentrations of up to 0.1 mg·L^−1^. The methodology for detection was described by Manukhov et al. [16] and Zavilgelsky et al. [13]. A 30-min pre-incubation of supernatant with bacterial culture was performed. “Control” samples contained, respectively, the unconditioned media corresponding to a specific strain (when estimating the activity of metabolites) and non-treated supernatant (when studying the influence of temperature, etc., on the activity of fermentates).

For luminescence measurements, an LM-01A automatic microplate luminometer (Immunotech, Prague, Czech Republic) was used. Measurements were carried out every 10 min for 120 min. The formula to evaluate the influence on RecA promoter expression was:

*I_s_* = (*L_e_*/*L_k_*) − 1 (1)

where (*I_s_*) is the induction factor; *L_k_* and *L_e_* are luminescence intensities of the control and experimental samples, respectively. A statistically-significant excess of *L_e_* over *L_k_* estimated with the *t*-criterion was considered as a sign of the significant influence on the induction effect.

Protective (anti-SOS) activity (*P*, %) was calculated taking induction into account in the presence of the corresponding protector concentrations:

*P* = (1 − (*I_p_*/*I_a_*)) × 100% (2)

where *I_p_* and *I_a_* are the induction factors of the SOS-response with the investigated influence in the presence of protector and in the control sample, respectively.

Statistical analysis was performed using Student’s *t*-test for *P* = 0.05. The calculations were performed using the method described in [14].

To study the thermostability of the cell-free supernatants, preparations were heated in a water bath to 85 °C. Proteinase and RNAse (Sigma-Aldrich, St. Louis, MS, USA.) were used according to the standard protocol [17].

### 2.4. Determination of the Parameters for Spontaneous and Induced Mutagenesis

An overnight culture of *E. coli* MG 1655 was diluted to a concentration of 3 × 10^8^–6 × 10^8^ CFU mL^−1^, then 5.0 μL of the suspension were added to 3 mL of LB as follows: (i) without addition of the inducer of mutagenesis or supernatant (control); (ii) with application of supernatant in a ratio of 1:9; (iii) with the addition of a ciprofloxacin solution at a concentration of 0.1 mg·L^−1^; and (iv) with the simultaneous administration of ciprofloxacin and supernatant at the indicated concentrations.

The concentration at which ciprofloxacin is an inducer of the SOS-response in bacteria was taken from previous studies [18,19]. The cultures were incubated at 37 °C for 24 h. Through the above-described preparation, a number of successive 1:10 dilutions of the culture in saline were prepared from the resulting cell suspension.

Surface inoculations were made with 100 μL culture on LB agar plates with and without the addition of rifampicin at a concentration of 70 μg·mL^−1^ according to the standard procedure [20]. Four plates for each sample were used. Colony counting on medium without antibiotics was performed after 48 h and on medium with rifampicin after 7 days.

The survival rate was calculated using the formula:

Survival, % = n_sample_/n_control_ × 100% (3)

where n_sample_ is the number of colonies after incubation with an inducer and n_control_ is the number of colonies after incubation without an inducer.

The mutant frequency was calculated by the formula:

Frequency = n_sample_/n_control_ (4)

where n_sample_ is the number of colonies on the medium with an antibiotic and n_control_ is the number of colonies on a medium without antibiotic.

The reliability of the mutagenic effect was evaluated by the statistical significance of the differences (*t*-test, *p* < 0.05) according to the number of colonies between the experimental and control groups, taking into account the dilution.

To confirm that the obtained colonies are true mutants, they were subsequently transferred to plates with rifampicin of the same concentration

## 3. Results

### 3.1. Biosensor Tests

Table 1 presents the results of screening with biosensors.

As a result of the study, it was found that:

- Six fermentates of *Lactobacillus* cultures (*L. rhamnosus*, *L. lactis* 6453, *Lactobacillus* sp. 6367, *L. acidophilus* 2647, *Lactobacillus* sp. 6379, *L. plantarum* B3242) showed the presence of potential antimutagenic properties;
- metabolites of *Lactobacillus* sp. 6367 (the average value of the protective effect is 66.36%) have the highest indicators of protective activity;

As can be seen from the data presented, the fermentates of both strains of *Bacillus* demonstrated SOS inhibitory activity (up to 54.21%) and the ability to reduce SOS-associated mutagenesis in bacteria. At the same time, the strain K1933 was characterized by a higher protective activity.

Compared with the protective effect that was observed with the strain of *Bacillus subtilis* KATMIRA1933, the supernatant of the strain *Bacillus amyloliquefaciens* B-1895 is more resistant to dilution and exhibits SOS inhibitory activity even when diluted 10,000-fold.

It was shown that an increasing dose of *Bacillus* fermentates correlates with an increase in the protective effect, as well, whereas for lactobacilli, the effect of dilution is not observed. In addition, the metabolites of lactobacilli in high doses exhibited a bactericidal effect. This may indicate that the target activity in different species of probiotic microorganisms is provided by different agents.

Trying to estimate the specific active factor in fermentates of bacilli, we treated them with temperature and ferments (proteinase K (SkyGen, Moscow, Russia) and RNase (Syntol, Moscow, Russia)). The active substance of the culture liquid, which has SOS inhibitory activity, does not react to temperature changes. Additionally, its activity under the action of both the proteinase and RNase, tested individually, does not show any statistically-significant decrease in the activity of both fermentates (the greatest difference between the control sample and ones treated by the above-mentioned factors was demonstrated for *B. amyloliquefaciens* B-1895 fermentate and was 1.6–2.75%).

### 3.2. Mutagenesis Assay

To confirm the data obtained in the biosensor tests, a mutagenesis assay was performed. Data on the activity of *B. amyloliquefaciens* B-1895 fermentate are presented in Table 2.

When transferred to plates with rifampicin of the same concentration, the obtained colonies of mutants successfully continued to grow under these conditions.

The fermentate of *B. amyloliquefaciens* B-1895 had no toxic effect on *E. coli* MG1655. The number of surviving cells in the medium supplemented with supernatant did not differ from the control. Furthermore, the supernatant did not protect cells from the toxic effect of ciprofloxacin: the number of surviving cells exposed to sub-lethal concentrations of ciprofloxacin did not differ significantly in the presence and absence of supernatant.

The fermentate did not induce amplification of mutagenesis in *E. coli* MG1655: the incidence of the occurrence of rifampicin-resistant mutants against background levels was not significantly different from the control; while at sub-lethal concentrations, ciprofloxacin was an inducer of mutagenesis in this system: the incidence of mutations increased more than three-fold (*p* < 0.05).

Thus, *B. amyloliquefaciens* B-1895 fermentate reduced the mutagenic effect of ciprofloxacin. Although the frequency of occurrence of resistant mutants by ciprofloxacin against the background of the supernatant was significantly higher than the control values (*p* < 0.05), it was half the rate of the appearance of resistant mutants by ciprofloxacin without the addition of supernatant (*p* < 0.05). Data on the activity of a second strain were similar and are not shown. This suggests that a decrease in the SOS response of bacteria under the action of the substances produced by these probiotic *Bacillus* strains leads to a significant decrease in the incidence of mutations causing antibiotic resistance in bacteria.

## 4. Discussion

The SOS response is, in short, a reaction of bacteria to DNA damage that activates error-prone DNA polymerases and is followed by amplification of mutagenesis; so-called “SOS mutagenesis” [21]. It consists of many stages and processes and involves a large number of genes that have been thoroughly described in a variety of papers [22,23,24].

As was mentioned before, RecA protein is a main target for inhibition as for DNA repair. RecA-like proteins are also highly conserved in bacterial species [25], which makes it a promising target in a wide range of potential pathogens. 

It seems that three are crucial points of the SOS-induced mutagenesis: ATP binding, ATPase activity and DNA strand exchange.

Alam et al. [2] characterized some inhibitors that block activation of the SOS-response induced by antibiotic by acting on RecA protein (phthalocyanine tetrasulfonic acid and others). It was also found that these inhibitors alter the activity of the quinolone, β-lactam, and aminoglycoside bactericidal antibiotics and reduces the acquiring antibiotic resistance mutations in bacteria or the transfer of mobile genetic elements that confer that resistance. Similar approaches have been proposed by Lee et al. [5]: substances that cause the formation of a dsDNA ligament with RecA, or inhibition of the binding of RecA to ATP; in particular, N^6^-(1-naphthyl)-ADP complexes were synthesized.

In one study [6], an effective system for screening inhibitors preventing ATP hydrolysis by RecA was proposed, based on a fluorescent assay. Five groups of substances were considered. As a result of screening for inhibitors of RecA’s ATPase activity, the authors reported that suramin and suramin-like substances possess such activity. 

This discovery was further developed by Nautiyal et al. [9]. The authors found that suramin can repress the SOS response induced by ciprofloxacin; and thus enhance the action of ciprofloxacin by inhibiting RecA activity at three previously-mentioned crucial points of the SOS-response. It was shown that the mechanism of action of suramin is likely due to conformational changes in RecA. However, a significant obstacle in using suramin may be connected to its negative charge: there is a doubt that suramin will be able to cross the cell walls of some bacteria, such as Mycobacteria.

Subsequently, the authors [3] have done an impressive job in conducting an in vitro screening of over 35,000 molecules for anti-SOS activity. The test was based on the detection of inhibition of the ATPase activity of RecA. The authors postulate that transition metal complexes, nucleotide analogs, structured peptides and polysulfated naphthyl compounds that have previously been identified as RecA inhibitors are either not cell-permeable or have been determined to have a pleiotropic effect on live bacteria. This has led them to search for more drug-like small molecule inhibitors of RecA.

In 2012, part of the same team conducted a widened screening of over 100,000 substances [7]. The most promising compounds identified in these screenings were then tested for the ability to potentiate the action of fluoroquinolones on *E. coli.*

In a similar screening process [8], computer simulations were applied to identify and develop substances with the necessary activity. Using genetic algorithms, the authors presented a method for the de novo design of potential inhibitors.

For the reasons stated above, selective RecA inhibitors are now considered as potential chemotherapeutic adjuvants to enhance antibiotic therapy. A study by Alam et al. [2] suggests that RecA can be inhibited by targeting the recruitment and polymerization of protein, by interfering with ATP binding or DNA binding. A complication that arises in this approach is that an ideal inhibitor of RecA should be specific for bacterial species. There is no doubt that for therapeutic purposes, we need a RecA inhibitor that selectively targets RecA in pathogens of interest without interfering with RecA homologs/orthologs in eukaryotes (such as gene Rad51). By using the metabolites of probiotic bacteria, this problem is no longer an issue, as the term “probiotic” itself implies that the metabolites of a given probiotic strain have been demonstrated as safe to the eukaryotic host.

The use of probiotic-derived metabolites, among other things, will eliminate the risks associated with the introduction of artificially-synthesized compounds, such as possible toxic, mutagenic and other deleterious effects.

There are quite a few studies on the antigenotoxicity of probiotic bacteria, including through the reduction of the SOS-response [26,27,28]. Nevertheless, in all available studies, a decrease in the SOS-response of bacteria is observed under the influence of fermentates of various probiotic bacteria. The chemical nature of inhibitors in these studies is not identified.

Recently, we demonstrated that fermentates of *Bacillus amyloliquefaciens* B-1895 and *Bacillus subtilis* KATMIRA1933 contain DNA protective and antioxidative substances [14]. The mechanism of this DNA-protective activity can be associated, in addition to the direct interception of oxidative radicals, with the switching off of the SOS-response by exogenous signals. In this study, we have applied a more specific system consisting of an inducer and biosensor specifically designed to react to the start of the first stage in the SOS-response.

Substances of a peptide nature [29] and/or signal RNA [30,31] are often key components of signaling networks within the microbial community. Therefore, the active factor was checked for thermostability and sensitivity to both proteinase and RNase. Judging from the data obtained, if the active substance is of a peptide nature, then they are not long molecules, but shorter oligopeptides.

Substances that provide the target activity in lactobacilli metabolites are still under study.

## 5. Conclusions

The strains described above, apparently, can be a source of metabolites with SOS inhibitory activity. Reasonable directions for further research are the identification of intermediates and genetic determinants of such activity and the selection of probiotic strains producing active substances for pharmacological purposes. This study demonstrates the potential of this approach in the search for naturally-derived SOS response inhibitors. Large-scale experiments on clinically-relevant pathogens will form the basis of our future research.

## Figures and Tables

**Table 1:** Protective effects of *Lactobacilli* fermentates on the *E. coli* MG1655 RecA-lux strain against ciprofloxacin.

**Strain**	**Anti-SOS Activity, %**						
Volume of fraction in solution, %	0.00001	0.0001	0.001	0.01	0.1	1	10
*Lactobacillus rhamnosus*	53.11 ± 1.23	64.39 ± 2.64	51.66 ± 3.16	64.51 ± 3.55	63.42 ± 3.78	62.39 ± 7.11	51.49 ± 5.44
*L. lactis* 6453	23.95 ± 1.01	17.89 ± 0.89	33.89 ± 2.72	35.21 ± 2.16	31.26 ± 1.76	19.39 ± 0.25	41.92 ± 3.32
*Lactobacillus* sp. 6367	69.08 ± 2.44	60.26 ± 3.19	64.80 ± 2.46	66.61 ± 6.66	63.86 ± 3.49	76.31 ± 6.93	63.62 ± 6.78
*L. acidophilus* 2647	48.36 ± 1.44	46.83 ± 2.26	48.16 ± 1.43	40.83 ± 3.82	53.18 ± 2.99	53.30 ± 3.24	Bactericidal
*Lactobacillus* sp. 6379	59.21 ± 2.86	54.22 ± 3.08	50.19 ± 2.39	54.14 ± 4,13	56.97 ± 3.04	57.31 ± 5.24	63.08 ± 5.93
*L. plantarum* B3242	33.50 ± 1.51	78.27 ± 4.11	44.99 ± 1.28	46.30 ± 3,78	75.21 ± 5.28	Bactericidal	Bactericidal
*Bacillus amyloliquefaciens* B-1895	0	0	2.20 ± 0.71	12.23 ± 1.23	35.24 ± 2.34	41.04 ± 3.75	54.21 ± 4.89
*Bacillus subtilis* KATMIRA1933	0	0	0	7.98 ± 1.09	26.84 ± 2.34	35.29 ± 4.96	50.84 ± 5.12
**Strain**	**Anti-SOS Activity, %**						
Volume of fraction in solution,%	0.00001	0.0001	0.001	0.01	0.1	1	10
*Lactobacillus rhamnosus*	53.11 ± 1.23	64.39 ± 2.64	51.66 ± 3.16	64.51 ± 3.55	63.42 ± 3.78	62.39 ± 7.11	51.49 ± 5.44
*L. lactis* 6453	23.95 ± 1.01	17.89 ± 0.89	33.89 ± 2.72	35.21 ± 2.16	31.26 ± 1.76	19.39 ± 0.25	41.92 ± 3.32
*Lactobacillus* sp. 6367	69.08 ± 2.44	60.26 ± 3.19	64.80 ± 2.46	66.61 ± 6.66	63.86 ± 3.49	76.31 ± 6.93	63.62 ± 6.78
*L. acidophilus* 2647	48.36 ± 1.44	46.83 ± 2.26	48.16 ± 1.43	40.83 ± 3.82	53.18 ± 2.99	53.30 ± 3.24	Bactericidal
*Lactobacillus* sp. 6379	59.21 ± 2.86	54.22 ± 3.08	50.19 ± 2.39	54.14±	56.97 ± 3.04	57.31 ± 5.24	63.08 ± 5.93
*L. plantarum* B3242	33.50 ± 1.51	78.27 ± 4.11	44.99 ± 1.28	46.30±	75.21 ± 5.28	Bactericidal	Bactericidal
*Bacillus amyloliquefaciens* B-1895	0	0	2.20 ± 0.71	12.23 ± 1.23	35.24 ± 2.34	41.04 ± 3.75	54.21 ± 4.89
*Bacillus subtilis* KATMIRA1933	0	0	0	7.98 ± 1.09	26.84 ± 2.34	35.29 ± 4.96	50.84 ± 5.12

**Table 2:** Antimutagenic activity of *B. amyloliquefaciens* B1895 fermentate in *E. coli* MG 1655 culture.

	Growth of Bacteria in Medium without Rifampicin			Growth of Bacteria on Medium with Rifampicin		
	Dilution	Average Number of Colonies per Plate	Survival Rate	Dilution	Average Number of Colonies per Plate	Mutation Frequency
Control	10^−6^	130 ± 15	100%	10^−1^	39 ± 6	(3.0 ± 0.3) × 10^−5^
Fermentate added	10^−6^	152 ± 11	117%	10^−1^	47 ± 9	(3.1 ± 0.3) × 10^−5^
Inducer added	10^−5^	220 ± 6	18% *	10^−1^	22 ± 7	(10 ± 0.2) × 10^−5^ *
Fermentate and inducer are added	10^−5^	228 ± 12	17% *	10^−1^	10 ± 2	(4.5 ± 0.3) × 10^−5^ *

* Significant difference from control, *p* < 0.05.
